# Transcranial Doppler-Based Surrogates for Cerebral Blood Flow: A Statistical Study

**DOI:** 10.1371/journal.pone.0165536

**Published:** 2016-11-23

**Authors:** Joseph Hart, Vera Novak, Charles Saunders, Pierre A. Gremaud

**Affiliations:** 1 Department of Mathematics, North Carolina State University, Raleigh, North Carolina, United States of America; 2 Department of Neurology, Beth Israel Deaconess Medical Center, Boston, Massachusetts, United States of America; 3 Engineering and the Environment, University of Southampton, Southampton, United Kingdom; Ehime University Graduate School of Medicine, JAPAN

## Abstract

It is commonly assumed that perfusion in a given cerebral territory can be inferred from Blood Flow Velocity (BFV) measurements in the corresponding stem artery. In order to test this hypothesis, we construct a cerebral blood flow (CBF) estimator based on transcranial Doppler (TCD) blood flow velocity and ten other easily available patient characteristics and clinical parameters. A total of 261 measurements were collected from 88 older patients. The estimator is based on local regression (Random Forest). Its performance is analyzed against baseline CBF from 3-D pseudocontinuous arterial spin labeling (pCASL) magnetic resonance imaging (MRI). Patient specific CBF predictions are of poor quality (*r* = 0.41 and *p*-value = 4.5 × 10^−12^); the hypothesis is thus not clearly supported by evidence.

## Introduction

Cardiovascular diseases are the number one cause of death globally: more people die annually from them (31%) than from any other cause [1]. Cerebrovascular diseases account for a significant proportion of these deaths–nearly 40% for stroke alone. Consequently, considerable efforts are devoted to the development of cerebral blood flow (CBF) measurement methods [2]. These methods include magnetic resonance imaging (MRI), positron emission tomography (PET), single photon emission computed tomography (SPECT), perfusion CT, xenon CT and others. The relative performance of CBF measurement methods has been extensively studied [3–14]. For instance, two distinct MRI protocols are considered in [15]: pseudocontinuous arterial spin labeling (pCASL, which is the MRI protocol used in the present study) and phase contrast; measurements from the two protocols are only weakly correlated (*r* = .59). The reported results are sometimes contradictory: [7] reports strong correlation (*r* = .89) between dynamic susceptibility contrast MRI and pulsed Arterial Spin Labeling (ASL), while [4] finds the results from these two protocols uncorrelated.

It is often difficult to synthesize and compare such studies because of

1. variable skill levels of the operators;
2. differences in size and homogeneity of patient groups;
3. population sizes that are too small to be statistically significant: among the previously cited studies, only two consider more than 35 subjects (152 subjects in [3] and 436 in [15]);
4. differences in equipment,
5. differences in the reported quantities and post-processing methods,
6. inconsistencies in elapsed times between repeated measurements,
7. differences in temporal vs. spatial resolutions across methods,
8. inherent variability of CBF even within the same subject [16].

Reflecting the above challenges, no specific protocol is currently considered as gold standard [17].

Under the assumption of a Poiseuille flow, an estimate of CBF can be obtained from the TCD measurement of the centerline velocity *v* through

$$CBF_{TCD}=\frac{\pi R^{2}}{M}\frac{v}{2\cos\theta },$$ (1)

where *R* is a characteristic value of the vessel radius, *M* the mass of the territory under consideration–here, the Middle Cerebral Artery (MCA) territory–and *θ* the insonation angle (i.e. the angle between the ultrasound probe and the vessel). The values of *M*, *R* and *θ* can, in principle, be estimated from medical images for each patient. In particular, *θ* can be approximated through elementary trigonometry using anatomical images, the location of the insonation site and the MCA location at the depth of insonation. Formula (1) illustrates in particular the importance of the radius of the stem artery *R* when attempting to use BFV as a surrogate for CBF. The quadratic dependency of the flow on the radius and the possibility of varying radii during measurement are at the origin of the most strident dismissals of BFV as a possible surrogate for flow, see for instance [18]. The extent to which the radius varies during experiments is itself somewhat controversial. While both [19] and [20] analyze the MCA diameter during hypercapnia and hypocapnia through similar methodologies, these studies significantly differ in their findings [17].

Yet, over the last five years, more than 1800 studies have relied on measurements of BFV for diagnostic evaluation of CBF, vasoreactivity to CO_2_ and blood pressure challenges. The underlying assumption is that vasoreactivity of small vessels and perfusion in the corresponding vascular territory can be inferred from BFV measurements in the stem artery. We demonstrate in this paper that this hypothesis is *not clearly supported by evidence*. Our approach consists in constructing a CBF estimator based not only on blood flow velocity (BFV) measured by TCD but also on other predictors that are easy to access in a clinical setting. The approximation process is based on methods from nonparametric statistics which can handle mixtures of categorical and numerical variables as well as noisy data. More specifically, we train Random Forest approximations on various subsets of the data to predict CBF as measured by pCASL-MRI.

## Materials and Methods

### Subjects

The results are based on data from 88 subjects (38 men and 50 women over 50 years of mean age of 67.7±8.02 years). Participants were recruited to participate in clinical trials (ADA 1-06-CR-25; NIH-NINDS 1R01-NS045745-01A2; NIH-NIA 1 R01- AG028760A2; 2009-2011) from community advertisement and through Beth Israel Deaconess Medical Center, Joslin Diabetes Clinic patient registries and the Harvard Cooperative Program on Aging research subject registry. All participants signed the informed consent form for the studies approved by the Institutional Review Board at Beth Israel Deaconess Medical Center (BIDMC), i.e., the Committee on Clinical Investigations (CCI). The participants provided consent to the use of their data in future research. The original protocols were amended to include analyses evaluating the relationships between TCD and MRI; the amendments were approved by the BIDMC CCI. The current retrospective analyses use exclusively de-identified datasets.

The data for this retrospective analysis of 88 subjects were selected from a database of records prospectively collected at the Syncope and Falls in the Elderly Laboratory and the Center for Magnetic Resonance Imaging at BIDMC. The database was composed of records from three completed projects spanning January 2002 to February 2008: Cerebral vasoregulation in the elderly with stroke (March 2003-April 2005); Cerebral vasoregulation in diabetes (January 2002-December 2005); and Cerebral perfusion and cognitive decline in type 2 diabetes (January 2006-December 2008) (see Acknowledgment/Funding Section for grant numbers). Cohort characteristics are summarized in Table 1. BFV and CBF measurements are, ideally, acquired on both the left and right side of the brain for each patient; left and right measurements are considered independently on each side and correspond to a total of 261 measurements. For most patients, 49 of them, one left and one right measurements are available. A total of 32 patients have two left and two right measurements. A full description of the number of available measurements is given in Table 2. Several patients had multiple measurements spanning several projects (i.e., several years); this makes it possible to investigate the predictive values of this type of data, see Results and Discussion below.

**Table 1:** Cohort chacteristics and distribution of healthy, hypertensive (HTN), diabetic (DM), diabetic and hypertensive (DM-HTN) subjects.

	total	male	female
participants	88	38	50
measurements	261	114	147
age	67.7±8.02	67.4 ±8.61	68.0±7.61
healthy	28 (31.8%)	16 (42.1%)	12 (24.0%)
HTN	41 (46.6%)	15 (39.5%)	26 (52.0%)
DM without HTN	2 (2.3%)	1 (2.6%)	1 (2.0%)
DM-HTN	17 (19.3%)	6 (15.8%)	11 (22.0%)

**Table 2:** Number of measurements (defined as either left or right MCA measurements) available per patient.

**number of patients**	1	49	1	32	1	3	0	1
**measurements per patient**	1	2	3	4	5	6	7	8

A complete dataset for each subject includes: demographic data, laboratory values, plasma hematocrit, TCD-based BFV in MCA, pCASL perfusion, blood pressure end tidal CO_2_ at baseline, during hypercapnia, hypocapnia and cognitive challenge; time of flight MR angiography (TOEF MRA) to characterize vessels in the Circle of Willis (CoW) and T1 and T2 weighted images to characterize brain tissue volumes. TCD recordings were visually inspected for signal quality before including in the analysis. Baseline MCA BFV and baseline CBF from 3-D pCASL were acquired on the same day. Patients were on a no caffeine diet 24 hours before the exam. Antihypertensive medications were tapered and stopped on the day of the study; medications not affecting the cardiovascular system were allowed. The TCD and MRI protocols are discussed below.

Inclusion criteria were: (i) *Healthy normotensive group*: normotensive (BP under 140/90 mm Hg); not being treated for any systemic cardiovascular, renal, or neurological disease; no focal deficit on neurological exam; normal glucose and hemoglobin A1c. (ii) *Hypertensive (HTN) group*: BP over 140/90 mm Hg or diagnosed/treated for hypertension. (iii) *Diabetes (DM) group:* diagnosed and treated for type 2 diabetes mellitus for more than one year. (iv) *DM-HTN group*: with both hypertension and type 2 diabetes. Exclusion criteria were: type 1 DM, major event or surgery within 6 months, stroke history, carotid stenosis, intracranial stenosis, uncontrolled HTN, significant renal, liver, cardiac disease or failure, substance abuse, body mass index over 40, failed TCD insonation.

### TCD protocol

About an hour after a light breakfast, participants were instrumented (PMD150 Spencer Technologies, Inc) with continuous monitoring of BFV in major Circle of Willis’ (CoW) vessels (anterior and middle cerebral arteries, ACAs and MCAs); continuous cardiovascular monitoring (beat-to-beat blood pressure, ECG), respiratory tidal volume, flow rate O_2_ and CO_2_ were simultaneously sampled at 500Hz using Labview. Blood pressure was measured beat-to-beat noninvasively from the finger using a volume-clamp method with a Portapres device (Finapres Medical Systems BV, Amsterdam, The Netherlands). TCD assessments were performed by an experienced sonographer (V.N.). Vessels were insonated bilaterally to record the maximum velocity for each vessel; probes were stabilized with 3D holders. A transtemporal window was used with sample volume set up in the Doppler mode, see Spencer Technologies and [21]. Average insonation depth was 54–57mm. The mean BFV *v* was calculated as an integral over time of the velocity envelope; systolic and diastolic peak velocities were recorded. Six minute supine baseline was acquired for each vascular territory. Insonation failure incidences were below 10%. Upon completion, participants were escorted to the MRI suite for MRI study.

### MRI protocol

CASL [22–26] is one of several Arterial Spin Labeling MRI techniques; it allows the non-invasive measurement of regional perfusion. It is based on electromagnetically labeling arterial blood water in the supplying vessels to an area under study. The CASL protocol uses “continuous” radio frequency pulses (≈ 2 seconds). After some time, the area is imaged; labeled and control states are compared to infer perfusion.

A GE 3 Tesla HDxt scanner with 8 channel brain coil was used. 3D CASL images were acquired with pseudo-continuous labeling, background suppression, and a volumetric stack of spirals fast spin echo acquisition. The resulting pseudocontinuous ASL (pCASL) [27] is a hybrid approach developed in collaboration with GE Healthcare. Modifications to the standard sequence include (i) interleaved labeling and background suppression [28] to enable longer labeling and better signal and (ii) transit time prescan [29, 30] to improve quantification of flow in subjects with vascular pathology or slow flow. Labeling was done at the level of cervical vertebra C1 for whole brain images and at the ICA level for vessel specific labeling [29]. Perfusion and vasoreactivity data were acquired through established protocols and methods [27, 29, 31, 32].

**Fig 1:**
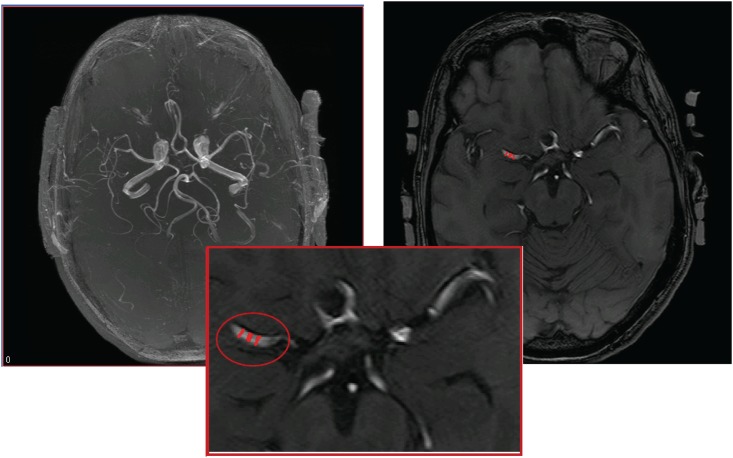
Left: time-of-flight MRA 3-D reconstruction; right: single-slice transverse view; bottom: zoom on the locations of three MCA radius estimates.

High resolution anatomical images were acquired through 3D magnetization prepared rapid gradient echo (MP-RAGE) and fluid attenuation inversion recovery (FLAIR). Perfusion images were averaged during each condition (6 minutes baseline normocapnia) to improve the signal-to-noise ratio. Perfusion and anatomical MR images (MP-RAGE and FLAIR) was co-registered to a standard template of regional vascular territories and segmented to calculate regional perfusion, gray matter, white matter, and cerebrospinal fluid volumes using the statistical parametric mapping software package (SPM, University College London, UK) [33] and tools written in IDL. ECG, end tidal O_2_, CO_2_ and blood pressure were simultaneously acquired. An anatomical template (Laboratory of Neuro Imaging, University of California, Los Angles, USA) was applied to measure gray matter (GM), white matter (WM) and intracranial volume (ICV). Vessel diameters were calculated from 3D MR angiography (time of flight, TOF) using the Medical Image Processing, Analysis, and Visualization (MIPAV) software from the Biomedical Imaging Research Services Section, NIH, Bethesda, MD, at 3 locations and averaged, see Fig 1. Based on image resolution, the accuracy of the resulting MCA diameters is conservatively estimated at ±0.4 mm.

### Predictor and response variables

We consider the pCASL-MRI CBF measurements *y*_*i*_, *i* = 1, …, *N*, where *N* is the number of observations, as response variables. The other factors *x*_*i*_ = (*x*_*i*,1_, …, *x*_*i*,14_), *i* = 1, …, *N*, are predictor variables, namely hematocrit (HCT), intracranial volume (ICV), weight, height, grey matter to ICV fraction (GM/ICV), white matter to ICV fraction (WM/ICV), body mass index (BMI), age, head length (front to back), diabetes (Y/N), hypertension (Y/N), gender, TCD BFV and MCA diameter.

To lower the dimension of the problem and hence help prediction, we only retain one predictor from each group of strongly correlated variables. More precisely, the analysis below does not include GM/ICV or WM/ICV as they are strongly correlated to ICV, neither does it include weight which is determined by BMI and height. The 11 considered predictors are thus HCT, ICV, height, BMI, age, head length, diabetes, hypertension, gender, TCD BFV and MCA diameter. Statistical information about them is presented in Tables 1, 3 and 4. A previous version of this study included all 14 predictors; the difference between the two sets of results is barely detectable.

**Table 3:** Mean values, standard deviations, and number of measurements of the considered numerical variables which are independent of side (for age, see Table 1).

variable	healthy	diseased
height (m)	1.67±0.0954 (83)	1.65±0.0934 (49)
BMI (kg/m^2^)	25.9±4.04 (83)	27.3±5.24 (49)
head length (dm)	1.90±0.106 (82)	1.92±0.171 (48)
ICV (ml)	1547±211 (80)	1634±248(49)
HTC % (dimensionless)	40.6±3.50 (83)	39.5±3.12 (49)

**Table 4:** Mean values, standard deviations, and number of measurements of the considered numerical variables which are side dependent.

**variable**	**healthy left**	**healthy right**
velocity (cm/sec)	40.6 ± 13.5 (63)	38.9 ± 15.9 (65)
diameter (mm)	2.35 ± 0.388 (58)	2.33 ± 0.309 (57)
CBF (ml/min/100g)	40.7 ±9.27 (84)	40.3 ± 8.86 (81)
	**diseased left**	**diseased right**
velocity (cm/sec)	41.9 ± 11.9 (40)	39.2 ± 14.0 (38)
diameter (mm)	2.48 ± 0.261 (25)	2.50 ± 0.258 (25)
CBF (ml/min/100g)	37.8 ± 9.25 (48)	37.0 ± 10.2 (48)

### Random Forests

The above dataset involves noisy measurements, correlated variables and both categorical and numerical inputs. To handle all three difficulties, we base our approach on Random Forests (RF) [34, 35] the principle of which we briefly recall. To describe a RF, we must first introduce regression trees [36, 37] which are essentially piecewise constant approximations based on the given data.

**Fig 2:**
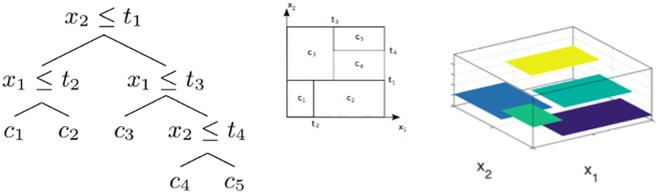
Illustration of a regression tree for a response depending on two predictors *x*_1_ and *x*_2_. Left: tree description of the approximation of the data using five constant values *c*_*i*_, *i* = 1, …, 5; middle: geometrical depiction of the tree; right: graph of the approximating function.

Fig 2 illustrates the construction of a regression tree for a generic problem with two predictors only. The first step of the algorithm consists in dividing the parameter space into two subdomains along either *x*_1_ = *t*_1_ or *x*_2_ = *t*_1_; the split-point *t*_1_ and the split variable, *x*_1_ or *x*_2_, are chosen so that approximation by constants on each side of *t*_1_ results in as small an error as possible. As the best constants are the mean values in each subdomain, the split point is taken so that each subdomain contains data points as similar as possible to each other. This simple recursive partitioning process is then repeated until a stopping criterion is satisfied (for instance, minimum number of data points in each subdomain [35]).

In general, the parameter space is partitioned into *K* “hyper-rectangular” regions *Ω*_*k*_, *k* = 1,…, *K*, in a space that has the dimension of the number of predictors. The response function is approximated by $y\approx T\left(x\right)=\sum_{k=1}^{K}c_{k}\;\chi_{k}\left(x\right)$ where *x* stands for the predictor variables, *χ*_*k*_ is the indicator function of *Ω*_*k*_ and *c*_*k*_ is a simple local model which is usually, as above, taken as the mean response in the leaf *Ω*_*k*_, i.e., $c_{k}=1/\left|I_{k}\right|\sum_{j=1}^{|I_{k}|}y_{j}$, *I*_*k*_ = {*j*; *x*_*j*_ ∈ *Ω*_*k*_}.

While regression trees are fast, simple and robust to irrelevant and/or missing variables, they may suffer from low accuracy and instability as small dataset changes can result in large tree changes. By considering ensembles of trees, RFs [34, 35] partially alleviate these problems. In addition, RF based models allow for correlated parameters, nonlinear interactions, mixed data type (categorical and continuous) and can handle missing data in a natural way (see below). A RF model consists of an ensemble of trees $\left\{T_{t};t=1,\ldots ,T\right\}$, each tree being grown from a bootstrap sample of the *N* data points. The process is outlined in Algorithm 1. We take T=500, *m* = 3, *n*_min_ = 5 and *p* = 11 in the results below.

**Algorithm 1** Random Forest

**function** RF(*x*, *y*, T, *m*, *n*_min_)

**Output:** tree ensemble ${\left\{T_{t}\right\}}_{t=1}^{T}$

 **for** t=1:T **do**

  draw a bootstrap sample with replacement of size *N* from the data (*x*, *y*)

  grow a tree *T*_*t*_ from the bootstrapped data, i.e.

  initiate domain *R* so as to contain all data

  **for** all terminal nodes *R* with |*R*| ≥ *n*_min_ **do**

   randomly select *m* variables among the *p* available variables

   determine the best split among these *m* variables

   split *R* into two daughter nodes

  **end for**

 **end for**

**end function**

To make a prediction at a new point *x*, the values of each of the T trees constructed above are averaged there

$$F\left(x\right)=\frac{1}{T}\sum_{t=1}^{T}T_{t}\left(x\right).$$ (2)

We refer to [35, 38] for additional information about implementation and the treatment of mixed data.

The data set contains *N* = 261 observations. Only 134 of these observations are complete. The imputation of missing values is an important part of data analysis and is the object of current research [39]. The flexibility of RF methods is here again an asset. We follow the iterative imputation scheme from [40] which has been shown to perform well even for mixed data. More precisely, let ***ξ*** be the *N* × (*p* + 1) data matrix, i.e.,

$$\xi =\left[\xi^{\left(1\right)},\cdots ,\xi^{\left(p+1\right)}\right]=\left[x_{i,1},\cdots ,x_{i,p},y_{i}\right],\;i=1,\cdots ,N.$$

For the *s*-th variable *ξ*^(*s*)^, *s* = 1,…, *p* + 1, we denote by $i_{mis}^{\left(s\right)}\subset \left\{1,\ldots ,N\right\}$ the indices of the missing values in that column and by $i_{obs}^{\left(s\right)}=\left\{1,\ldots ,N\right\}\backslash i_{mis}^{\left(s\right)}$ the rest of the indices. The data matrix *ξ* is then partitioned in four parts

1. the observed part of *ξ*^(*s*)^: $\zeta_{obs}^{\left(s\right)}=\xi^{\left(s\right)}\left(i_{obs}^{\left(s\right)}\right)$,
2. the missing part of *ξ*^(*s*)^: $\zeta_{mis}^{\left(s\right)}=\xi^{\left(s\right)}\left(i_{mis}^{\left(s\right)}\right)$,
3. the part of ***ξ***, without the *s*-th column, corresponding to $i_{obs}^{\left(s\right)}$: $\eta_{obs}^{\left(s\right)}=\left[\xi^{\left(i\right)}\left(i_{obs}^{\left(s\right)}\right)\right]$, *i* = 1,…, *s* − 1, *s* + 1,…, *p* + 1,
4. the part of ***ξ***, without the *s*-th column, corresponding to $i_{mis}^{\left(s\right)}$: $\eta_{mis}^{\left(s\right)}=\left[\xi^{\left(i\right)}\left(i_{mis}^{\left(s\right)}\right)\right]$, *i* = 1,…, *s* − 1, *s* + 1,…, *p* + 1.

The algorithm is initiated by making initial guesses for the missing values of ***ξ*** through mean imputation. Then, for each *ξ*^(*s*)^, a RF is learned with predictor $\eta_{obs}^{\left(s\right)}$ and response $\zeta_{obs}^{\left(s\right)}$; $\zeta_{mis}^{\left(s\right)}$ is then predicted by applying RF to $\eta_{mis}^{\left(s\right)}$ and ***ξ*** is updated. The process is repeated till convergence of the data matrix.

## Results

**Fig 3:**
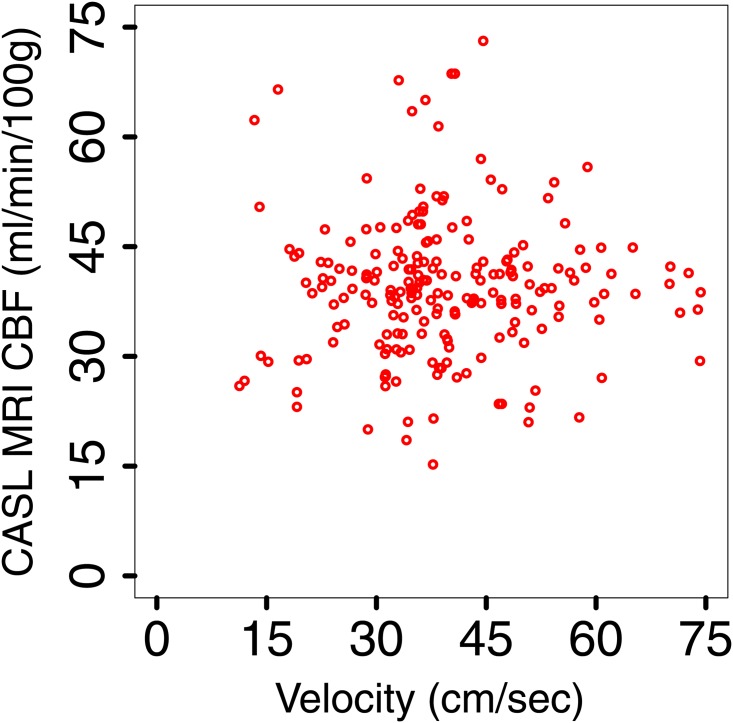
Scatter plot of the pCASL MRI CBF data versus TCD velocity.

The scatter plot of the pCASL MRI CBF data versus the TCD velocity measurements shows no correlation, see Fig 3.

A comparison between pCASL MRI CBF data and TCD CBF as computed from Eq (1) (for the 98 observations for which estimations of *M*, *R* and *θ* are available) shows that the two are uncorrelated (*r* = 0.13 and *p*-value of 0.25). We conclude that the TCD and MRI data are not linearly correlated.

We investigate the possible existence of more involved dependencies through local regression in the form of random forest models. Four numerical experiments are conducted corresponding to four different levels of MRI measurement accessibility in the model’s learning set. In each one, an ensemble of 500 trees is constructed through bootstrap sampling. The sets on which the RF is “learned” are as follows.

**Experiment 1:** All but one measurement: leave out one measurement cross-validation. This possibly includes multiple measurements for the same patient and the same side as the one being predicted.
**Experiment 2:** Leave out measurements from the same patient and the same side. This possibly includes measurements for the same patient but excludes data from the same side as the one being predicted.
**Experiment 3:** Leave out measurements taken at the same time. This possibly includes measurements taken from the same patient but at different times.
**Experiment 4:** All but one patient: leave out one patient cross-validation. No MRI measurements from a specific patient are used to predict CBF for that patient.

**Fig 4:**
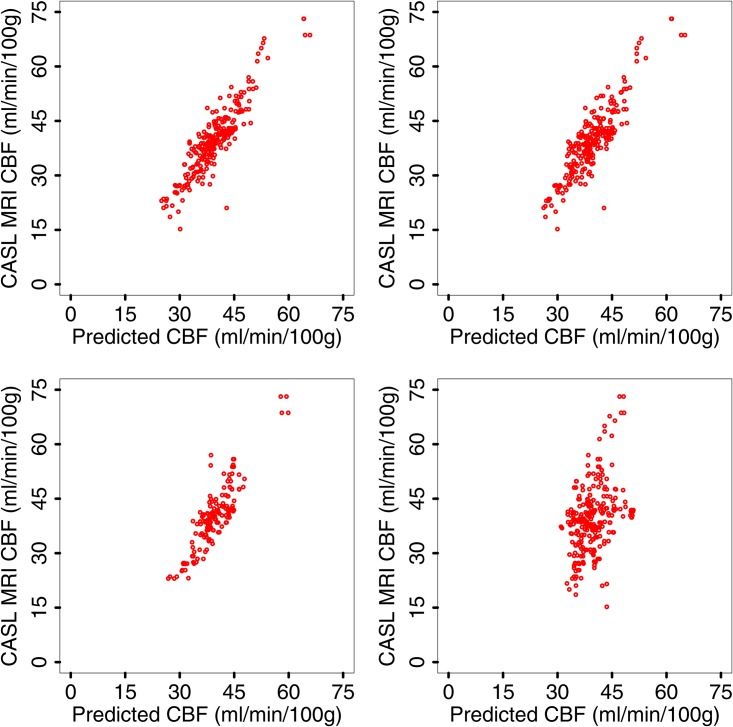
pCASL MRI CBF data versus predicted CBF. From left to right and top to bottom, experiments 1 to 4, see text; corresponding relative correlations are *r* = 0.91, *p*-value = 2.2 × 10^−16^, *r* = 0.89, *p*-value = 2.2 × 10^−16^, *r* = .88, *p*-value = 2.2 × 10^−16^ and *r* = 0.41, *p*-value = 4.5 × 10^−12^.

Fig 4 displays the resulting relationship between predicted and observed CBF.

## Discussion

The results from the above four experiments are summarized in Table 5.

**Table 5:** Correlations between predicted and measured CBF for the above four experiments. Due to cross-validation and the number of the various measurements available, see Table 2, the number of data points used to learn the models vary.

Experiment	Learning Data	r	p
1	260	.91	2.2 × 10^−16^
2	257-260	.89	2.2 × 10^−16^
3	259-260	.88	2.2 × 10^−16^
4	253-259	.41	4.5 × 10^−12^

In experiments 1 and 2, CBF in one of the MCA territories, left or right, of a specific subject is predicted using CBF measurements taken from that subject on the opposite side. Both experiments have only limited practical interest with the possible exceptions of asymptomatic cases with poor/no insonation on one side, those who cannot undergo MRI imaging due to metal implants or cases with transient ischemia or other unexplained unilateral symptoms that would have otherwise normal imaging studies but would require continuous monitoring. In both cases, the predictor is however remarkably accurate, indicating low intrapatient variability.

In experiment 3, we only use CBF measurements taken at a different time (i.e., the subject came to the clinic on multiple occasions). There are 162 such data points as some subjects only came to the clinic one time. The results indicates that there may be some predictive power in combining TCD with previous CBF measurements.

If no CBF measurement for a specific patient informs the predictor (experiment 4), the proposed CBF estimator only gives a poor CBF approximation for that patient, as displayed in Fig 4, bottom right. The discrepancy observed in experiment 4 casts doubt on the usefulness of TCD measured BFV as a viable surrogate for CBF.

The results of experience 4 do not improve significantly if the model is learned separately on the sets of healthy and diseased subjects (*r* = .48 and *p* = 2.2 × 10^−16^). It is striking, as mentioned above, that adding an approximation of the insonation angle to the list of predictors (these are available for 98 observations only) does *not* improve correlation between predicted and observed CBF. Indeed, the insonation angle can take values from 0 to up to 40 degrees; the corresponding geometric factor 1/cos(*θ*), see Eq (1), can thus induce changes larger than 40% in the velocity readings. The fact that such a potentially important factor does not seem to make a significant difference points to the possible noisiness of the data.

For TCD, causes of noisiness includes the quadratic dependence of the flow on the diameter *D*. We rewrite Eq (1) as $CBF_{TCD}=CD^{2}$, where $C=\frac{\pi }{4M}\frac{v}{2\cos\theta }$. Therefore, a relative measurement error Δ in the diameter, i.e., *D* ≈ *D*(1 + Δ), results in a relative error in surface area (and thus flow) of 2Δ + Δ^2^ ≈ 2Δ. In other words, a 10% relative error in diameter measurement, as is the case here and in [11], will potentially contribute over 20% of relative error in flow estimates. For MRI, the well-documented low signal-to-noise ratio [41] may contribute to noisiness of the pCASL-MRI measurements. Another potential source of noise is associated to difficulties in identifying perfusion territories. Indeed, perfusion measurements using pCASL in individual vascular territories are determined by co-registration of MR images to the standard template. There is, however, no accurate way to estimate the actual perfusion territory and collateral beds for a given patient. Imaging approaches that would specifically label MCA blood flow were not available in our protocol.

The above experiments display a noticeable bias as, for instance, a simple linear regression line would not intersect the origin in any of the four cases; this effect increases strongly as the amount of information used for inference diminishes from Experiment 1 to Experiment 4. Possible reasons for a systematic bias include the following.

1. *Underestimation of CBF through pCASL-MRI:* This may result from the arterial transit time being longer than the post-labelling delay and may be exacerbated by possible collateral flows [42]. pCASL perfusion measurements also strongly correlate with hematocrit; consequently, CBF may be underestimated in subjects with lower hematocrit (common in older people) [43].
2. *Incorrect characterization of the flow profile:* The predicted CBF relies on Eq (1) which results from the assumption of a Poiseuille flow, i.e., a flow where the longitudinal velocity *V* depends on the distance *r* to the centerline through $V\left(r\right)=v\;\left(1-\frac{r^{2}}{R^{2}}\right)$, *v* being the centerline velocity measured by TCD. This parabolic profile is an idealization; the actual longitudinal velocity *V* may be a non-monotonic time-dependent function of *r* and/or may not have a cylindrical symmetry [44].
3. *One or more key predictors may be absent from the considered dataset*.

The remarkable difference in accuracy between experiments 1-2 and 4 indicates that left and right MCA CBF are generally correlated for any given patient. Further analysis is needed to determine the predictive power of using CBF measurements from earlier visits to the clinic as in experiment 3; such a study would require additional data.

More generally, our experiments address the possibility of CBF prediction for a specific subject through data analytics by

- collecting easily available information about that subject–the above predictors,
- leveraging information, predictors and response, available for a group of “like-subjects” identified here through RF.

While such an approach would be an asset in the clinical setting in terms of both cost and convenience, the present results do not support its feasibility, at least for the set of predictors and response considered here.
